# Subthalamic deep brain stimulation versus best medical treatment: a 12-year follow-up

**DOI:** 10.1007/s13760-022-01874-8

**Published:** 2022-01-27

**Authors:** Alain Maertens de Noordhout, Micheline Mouchamps, Jean-Michel Remacle, Stéphanie Delstanche, Vincent Bonhomme, Michel Gonce

**Affiliations:** 1 University Department of Neurology, Hôpital de la Citadelle, 4000 Liège, Belgium; 2 Department of Neurosurgery, Hôpital de la Citadelle, 4000 Liège, Belgium; 3 Department of Anaesthesiology, Hôpital de la Citadelle, 4000 Liège, Belgium

**Keywords:** Parkinson's disease, Movement disorders, Deep Brain stimulation, Long-term follow-up

## Abstract

**Purpose:**

Electrical stimulation of the sub-thalamic nucleus (STN-DBS) is well established to alleviate motor fluctuations in advanced Parkinson’s disease but little is known about its very long-term efficacy.

**Methods:**

We followed over 12 years 15 parkinsonian patients having undergone STN-DBS and compared them to a matched group of 14 patients with best medical drug therapy. All had been considered as good candidates for surgery. They were allocated to each group depending on their own decision.

**Results:**

After 12 years, mortality rates were similar in both groups. In the DBS group, best “on” UPDRS III scores (on medications, on stimulation) remained significantly better and dyskinesia shorter and weaker than in the drug-treated group (on medication only). Yet, looking at independent life and quality of life (QoL) evaluated with PDQ39, no significant difference could be observed between groups at the end of follow-up, probably due to development of dopa- and stimulation-resistant motor and non-motor symptoms like falls, freezing, dementia, apathy and depression, the latter two more frequent in the DBS group.

**Conclusion:**

Drug- and DBS-resistant symptoms and signs occur more often after long disease evolution and in elder patients. It might be why differences in QoL between both groups no longer existed after twelve years as, compared to other studies, our patients were older at inclusion.

## Introduction

For nearly three decades after the original report of the Grenoble team [1], several studies have indicated that deep brain stimulation (DBS) of sub-thalamic nucleus (STN) or internal globus pallidus (Gpi) can dramatically improve motor and general condition of fluctuating parkinsonian patients [2, 3]. However, besides surgical risks such as intracerebral hemorrhage, these procedures can also have unwanted effects: apathy, cognitive disturbances, depression, impulse control disorders including suicide risk, speech problems, weight gain, poor balance, eyelid opening apraxia, etc. [4, 5]. Some of the above symptoms may not be directly due to DBS, but also the result of natural course of the disease and reduction of dopaminergic drugs.

Despite these, one first follow-up study of DBS patients showed that motor benefit, although slightly declining, remains significant up to 5 years after surgery [6]. This seminal study also indicated that STN-DBS apparently does not alter the natural course of the disease. Little is known however on the outcome of such patients after longer periods, compared to those who did not undergo surgery. A report by Aviles-Olmos et al. [7] indicated that STN-DBS was still effective in improving motor performances after 8–11 years, yet with progressive loss of quality of life due to increased axial signs, including falls, and intellectual decline. This was a retrospective study without a control group. Another study [8] compared evolution of a group of patients treated with STN-DBS versus best medical treatment over a relatively long period but range of follow-up was variable (4–11 years). A very recent monocentric retrospective analysis [9] of 51 STN-DBS patients followed up to 15 years after surgery indicates that time spent with dyskinesia and “off” condition remained significantly shorter than before surgery. However, there was no control group.

In the present study, we followed over 12 years, two similar cohorts of parkinsonian patients having or not undergone STN-DBS. Preliminary results have been presented before (JNLF Bordeaux, 2018).

## Patients and methods

Between 1997 and 2005, twenty-nine patients meeting UK brain bank criteria [10] for idiopathic Parkinson’s disease (15 men and 14 women aged 51–74, median 65, median disease duration: 9.7 ± 3.1 years) were screened as possible candidates for bilateral STN-DBS, based on the then recommended criteria for this procedure. Inclusion criteria were as follows: clear and long-lasting (> 5 years) response to levodopa, marked motor fluctuations despite fractionated levodopa doses, including disabling daytime “off” periods and/or troublesome dyskinesia. Normal brain MR and cognitive status for age, as well as absence of severe depression or delusion were also mandatory. All lived at home and were independent when “on”.

At that time, for reimbursement of DBS procedure and devices, a full medical report for each patient had to be submitted to a national commission of the Belgian health services (INAMI-RIZIV), including movement disorder specialists plus representatives of the administration. All 29 submitted cases were considered eligible for surgery. Health authorities also demanded a yearly report of evolution, which is no longer the case nowadays.

Possible benefits, risks and follow-up needs after surgery were explained to every patient by one neurosurgeon (MM) and one neurologist (AMdN). They were then divided on “intent to treat” basis into 2 groups, those choosing surgery and those preferring to stay on drug treatment. Thus, patients were not randomized but allocated to one arm or the other depending on their own decision. All gave written informed consent to the study. Publication of the results of this work was authorized by the local Ethics Committee (B4122021000032).

Patients were followed over 12 years, when final evaluation was completed. At least one visit per year was required. Additional non-drug therapies (physiotherapy, sports, etc.) were allowed and recommended in both groups. A patient missing 1 visit over 2 years was considered as a drop-out. It did not happen except for other reasons (see below).

Patients’ characteristics at inclusion are summarized in Table 1. The DBS group (*n* = 15) was slightly younger (median age 63) compared to the 14 drug-treated (median age 66, not significant: *p* = 0.28, Mann–Whitney), but they were otherwise comparable for mean levodopa dose (775 vs 750 mg/day), use of dopamine agonists converted into levodopa equivalents to calculate a global levodopa equivalent daily dose (LEDD) [11], disease duration (10.1 vs 9.4 years), UPDRS and Schwab and England “off” and “on” scores.

**Table 1:** Patients’ characteristics at inclusion

Patients	DBS group (*n* = 15)	Drug-treated group (*n* = 14)	Difference at inclusion (Mann–Whitney)
Age (years)	63 ± 6.4	66 ± 7.1	*p* = 0.24
Disease duration (years)	9.4 ± 5.2	10.1 ± 6.3	*p* = 0.7
Best UPDRS III	19 ± 8.5	20.2 ± 13.5	*p* = 0.78
Worst UPDRS III	57 ± 12.2	54.2 ± 15.5	*p* = 0.59
UPDRS-IV (dyskinesia)	8.4 ± 2.9	7.9 ± 3.3	*p* = 0.67
Best Schwab and England	93 ± 7	89 ± 11	*p* = 0.25
Worst Schwab and England	43 ± 19	51 ± 16	*p* = 0.23
Dopa equivalents/day (mg)	1115 ± 158	1070 ± 137	*p* = 0.8

DBS patients were implanted in 3 different centers, as we only initiated this type of surgery in 1999 (3 in Grenoble, 1 in Lille, and 11 locally). The same procedure was used in all centers (5 microelectrode millimetric recordings, intra-operative testing with awake patients). Only 3 PD patients implanted in Grenoble underwent ventriculography. In others, STN location was determined using 1.5 Tesla magnetic resonance imaging (MRI) scans. Implanted electrodes were the same in all patients who underwent STN-DBS (Medtronic^®^3389). In the days following intervention, computerized tomograms (CT) of the brain were performed to ensure correct placement of the electrodes and rule out hemorrhagic complications. At that time, we did not use post-operative MRI as it was not yet approved for Medtronic^®^ DBS implantable devices.

Motor scores (UPDRS III, on stimulation, on medication) at 1 year were not different for patients implanted in all three centers, which allowed us to conclude that surgical procedures were conducted in similar ways in all three centers. Subsequent follow-up was ensured in our center, though some extra visits could be proposed in foreign implanting centers. All 14 drug-treated were followed locally. No direct or delayed complication of surgical procedure was noted, except in one patient who showed infection of the battery after 3 months, displaced to another site (abdomen instead of pectoral region).

The patients of both groups were evaluated as follows. They were asked to attend the outpatient clinic in the morning, 30–60 min after previous drug intake and stay until they felt in their worst daily condition. This could sometimes take as long as 4–6 h. Schedule of drug administration was not modified during the observation period. Thus, best and worst UPDRS III ratings were performed “on medications” and “on DBS” only (in the DBS group). Indeed, adding other conditions like switching “off” DBS or skipping one drug dose would have prolonged visits unduly or require hospitalization, which was not in the study protocol and would have hardly been accepted by patients.

In the DBS group, implanted devices needed periodic replacements with slightly different batteries, though without significantly changing stimulation parameters over the whole observation period (for instance Itrel II > Kinetra > Activa, Medtronic^®^). Only minor alterations (± 0.2 V from parameters at 6 months after surgery) of stimulation intensities were allowed, either by the physician or the patient. This option was chosen because at the time patients were implanted, it was still unknown whether DBS modifies the natural evolution of Parkinson’s disease. Nevertheless, after 2006, we tried for brief periods low-frequency (50 Hz) stimulations in 3 patients with resistant freezing, without significant benefit and re-instated initial parameters.

After 12 years, the same parameters described in Table 1 were measured, plus quality of life (QoL) using the Parkinson’s disease questionnaire (PDQ-39) [12], not yet widely used when first patients were included (some of them before 2000). UPDRS III and IV scores were measured “on stimulation, on medications” in the DBS group. As stated above, for practical and ethical reasons, it was not possible to test DBS patients “off-DBS, off-drugs”.

Also, we interviewed patients and/or caregivers about these items: were they still alive, living at home or not, remained independent for activities of daily living (ADL), developed dementia or other non-motor symptoms such as depression and apathy. These were scored on items 17–29 and 31–36 of PDQ-39.

Comparisons between groups were made using Mann–Whitney *U* test for non-parametric distribution (Tables 1, 2) and for longitudinal follow-up of DBS patients, two-tailed analysis of variance (ANOVA) with post hoc Bonferroni corrections for repeated time measurements (Table 3). Descriptive statistics of the 11 DBS patients at end of study suggested a normal distribution for age, disease duration, LEDD and DBS stimulation parameters, in spite of limited size of sample.

**Table 2:** Evaluation of remaining patients after 12 years

	DBS (*n* = 11/15)	Drug-treated group (*n* = 9/14)	Statistical differences
Age at final evaluation	74 ± 8.2	76 ± 7.1	*p* = 0.8
Deaths and causes	4 Cancer (2) Heart failure (2)	3 Cancer (1) Inhalation pneumonia (2)	
Best UPDRS III	24.2 ± 11.2	29.3 ± 10.3	***p* = 0.03**
Worst UPDRS III	62 ± 13.1	65 ± 11.1	*p* = 0.6
UPDRS-IV (dyskinesia)	3.9 ± 1.1	6.3 ± 1.3	***p* < 0.001**
Percentage daytime “off”	28 ± 14	38 ± 15	*p* = 0.15
Best Schwab and England	74 ± 14	72 ± 11	*p* = 0.8
Worst Schwab and England	43 ± 11	39 ± 13	*p* = 0.5
LEDD (mg)	655 ± 132	845 ± 128	***p* = 0.03**
PDQ-39 scores at end of study (raw)	32.2 ± 11.2	35.8 ± 14.8	*p* = 0.5
Patients still at home	7/11	5/9	*p* = 0.8

Significant differences in bold, Mann–Whitney

In the DBS group, measurements made “on stimulation, on medications”, in the drug-treated group, measurements made “on medications”

**Table 3:** Evolution over time in the 11 patients of the DBS group who completed the study

	Pre-operatively meds only	6 months	6 years	12 years	Difference pre-op—6 months	Difference pre-op—12 years
Best UPDRS III on meds, on DBS	17 ± 8.2	17.5 ± 5.6	21.3 ± 7.8	24.2 ± 11.2	*p* = 0.92	*p* = 0.17
Worst UPDRS III on meds, on DBS	54 ± 9.92	28.8 ± 7.3	31.1 ± 8.3	62 ± 13.1	***p* = 0.01**	*p* = 0.92
LEDD mg	1032 ± 144	483 ± 86	552 ± 76	655 ± 132	***p* < 0.001**	***p* = 0.01**

Significant differences in bold, two-tailed ANOVA + post hoc Bonferroni corrections for repeated time measurements. See text for details of patients’ conditions

## Results

At final assessment, 11/15 patients were still alive in the DBS group and 11/14 in the drug-treated ones. Two of the latter withdrew during follow-up to start with jejunal levodopa infusion (Duodopa^®^). Patients who shifted to Duodopa^®^ did not differ from the rest of the “drug-treated” group except for more severe dyskinesia. They withdrew after 6 and 8 years of follow-up respectively (aged 72 and 75 years, disease duration 12 and 15 years). None was lost to follow-up except the latter two, as Duodopa^®^ infusions were managed by another team in another hospital, with very frequent visits. This might have altered the course of the disease compared to patients under oral regimen. This is why we excluded them from the study.

No death was directly linked to PD or complications of surgery in the DBS group but two died from lung infection in the drug therapy group (see Table 2). Both occurred late in the follow-up (8 and 9 years, ages 74 and 81, 14 and 17 years of disease duration) and these patients had developed refractory swallowing problems over time.

Two (unsuccessful drownings) suicide attempts were reported in the DBS group in the first 3 months after surgery (patients aged 54 and 58). They had developed impulse control disorders soon after DBS, while no such problems were present before surgery, despite both were under dopamine agonists. Those had been discontinued progressively before implantation. Levodopa daily doses (LEDD) at time of suicide attempts were low in both (300 and 150 mg/day respectively). Brain CT indicated that active contacts of electrodes were correctly located inside the sub-thalamic nucleus, though with less precision than with MRI, not routinely used at that time. Yet, motor improvement in both argued against misplacement of leads.

Number of patients still at home at 12 years were similar in both groups (7/11 and 5/9). Reasons for moving to nursing homes were dementia (4/11 and 3/9), behavioral disturbances, frequent falls, incontinence or death of spouse.

Despite significant differences in LEDD between groups (see Table 2), PDQ-39 scores were not strikingly different after 12 years, although DBS patients had significantly less dyskinesia and shorter “off” periods, though not reaching statistical significance. This point is noteworthy as PDQ-39 leaves the largest part to motor items. Interrogating patients, family and caregivers, poor scores were mainly due to the following: levodopa-resistant freezing episodes with falls, behavioral problems (often at night), lack of initiative in DBS patients, delusion and dementia (mainly items 17–29 and 31–36 of PDQ-39, but also orthopedic problems linked to falls.

Levodopa-equivalent daily doses (LEDD) have been reduced over time, not only in the DBS group, but also in drug-treated patients. This was mainly due to withdrawal of DA agonists in people having developed behavioral problems or dementia.

In Table 3, we present the evolution over time of core parameters measured in the 11 patients of the DBS group who completed the study. Deterioration of motor scores mostly occurred after 6 years or more. Such interim parameters have not been systematically measured in the drug-treated group because we were mostly interested in quality of life over a long observation period.

## Discussion

Compared to patient population with long follow-up reported so far, our cohort was somewhat different, mostly because median age was significantly higher than in previous studies [2, 3, 6]. This might cause a bias toward earlier occurrence during follow-up of late motor and non-motor complications of PD (dementia, refractory axial disorders) but also other illnesses, for instance orthopedic. Such problems have an impact upon QoL and especially ability to remain independent at home. They should be comparable in both arms, as they were statistically comparable at study onset.

To our best knowledge, we found no published data of long-term outcomes of matched PD cohorts eligible for DBS, comparing those having undergone STN-DBS and those choosing to remain under best drug therapy, with the limitation that our groups were small. One might argue that DBS patients were more closely followed or had stronger motivation to comply with study program. It was not the case, except in the first 6 months after surgery, which required some additional visits for stimulation adjustments after disappearance of temporary “lesional” effects of electrode positioning.

Like Krack et al. [6] we observed a clear-cut reduction of “off” period duration and troublesome dyskinesia in the DBS arm of our study, which lasted up to 12 years after study onset, even if fading on the long term. Also, LEDD remained significantly lower in the DBS group.

What interested us most was quality of life of patients and caregivers over time.

From PDQ-39 scores plus patients’ and caregivers’ interviews, it appeared that after 12 years, levels of independence were not strikingly different between DBS and “drug” groups, despite better motor scores in the DBS group. Clearly, this was caused by the development over time of motor and non-motor dopa- and DBS-resistant symptoms like falls, and dementia, plus apathy and depression in the DBS group. A recent study [13]) also showed that axial symptoms predicts mortality in Parkinsonian patients with subthalamic stimulation. One might argue that optimization of DBS parameters should have yielded better results than in our cohort but, over the 12-year period, attempts of adjustments did not provide significant improvement in the remaining patients. This was also reported by others [9].

In a new retrospective analysis of the Grenoble cohort [9], Bove and colleagues reported a sustained benefit of STN-DBS beyond 15 years in 51 patients, not only on motor function but also on global quality of life (QoL). One explanation for this discrepancy was the much younger age of their patients at the time of surgery (51 ± 8.5 years old), even if the disease duration was similar (11.3 ± 3.8 years) to ours. Interestingly, 35% of their patients have developed dementia, usually more than 10 years after surgery. This is not very different from what is generally reported in the natural evolution of the disease. Also, that study reported results of only 51/138 patients who underwent STN-DBS in Grenoble, while 56 were lost to follow-up and 31 had died from various causes. One might argue that those might have been more severely affected than patients who were evaluated.

This study and other [7, 9] might be an argument for use of deep brain stimulation in younger patients and maybe earlier stages of disease [14], as clearly dopa- and DBS-resistant symptoms develop not only after long disease duration, but also with advancing age. Considering the overall cost of DBS, careful selection of eligible patients seems of utmost importance.
